# Nutraceuticals Against Oxidative Stress in Allergic Diseases

**DOI:** 10.3390/biom15091347

**Published:** 2025-09-20

**Authors:** Marilena Di Salvo, Alessandra Ventre, Enrica Dato, Marco Casciaro, Sebastiano Gangemi

**Affiliations:** 1 Department of Clinical and Experimental Medicine, Unit and School of Allergy and Clinical Immunology, University Hospital of Messina, University of Messina, 98125 Messina, Italy; marilenadisalvo98@gmail.com (M.D.S.); alessandraven144@gmail.com (A.V.); enricadato98@gmail.com (E.D.); gangemis@unime.it (S.G.); 2 Department of Medical Sciences, Unit and School of Allergy and Clinical Immunology, University Hospital of Messina, 98125 Messina, Italy

**Keywords:** oxidative stress, nutraceuticals, allergies, ROS, asthma, atopic dermatitis, rhino-conjunctivitis, food allergies, exogenous antioxidants

## Abstract

Antioxidant mechanisms consist of both enzymatic and non-enzymatic compounds, which can be either endogenous or exogenous and play a crucial role in counteracting oxidative stress. These compounds are primarily obtained through the diet. Vegetables, plants, and fruits contain a wide range of alkaloids, polyphenols, and terpenoids, collectively referred to as “phytochemicals.” Many of these substances are responsible for the beneficial properties of fruits and vegetables, which are essential components of a healthy lifestyle, contributing to the prevention of chronic diseases and the promotion of longevity. Nutraceuticals are bioactive substances present in food—or its components—that exert positive effects on health and may help prevent or treat various disorders. In this review, we examine the main applications of nutraceuticals in allergic disorders. The literature reports numerous studies on exogenous dietary antioxidant supplementation in various allergic conditions, including bronchial asthma, atopic dermatitis, food allergies, allergic rhino-conjunctivitis, urticaria, and angioedema. In some of these conditions, promising results have been observed. These positive outcomes are generally associated with a reduction in oxidative stress markers, enhancement of antioxidant systems, and, in some cases, anti-inflammatory effects. The administration of exogenous substances through food derivatives or dietary supplements, when scientifically standardized, has been proven to be effective. However, further large-scale, unbiased studies are needed—particularly those that include a broader range of oxidative stress biomarkers.

## 1. Introduction

Reactive oxygen species (ROS) are byproducts of our cells’ metabolism and they are mostly represented by superoxide anion radicals, hydrogen peroxide, and hydroxyl radicals. ROS production is considered a natural consequence of cellular metabolism, but it can also be triggered by exposure to external phenomena, and their overproduction could threaten our homeostasis because they could damage DNA [1,2,3,4].

Our cells survive without major damage as a result of the fine balance between ROS production and defense mechanisms, known as antioxidant processes (AOX), which mainly comprise, among others, endogen enzymes such as superoxide dismutase (SOD) catalase (CAT), glutathione peroxidase (GPx), and myeloperoxidase [5,6,7,8].

Each endogenous enzyme has a specific function: superoxide dismutase works by catalyzing the dismutation reaction of 02, resulting in the formation of 02 and H202; catalase and glutathione peroxidase decompose H202 into H20 and 02; myeloperoxidase catalyzes hypochlorous acid (HOCI) production [9].

This equilibrium could be compromised, perhaps due to overexposure to toxic factors, resulting in an ROS overload that can participate in the pathogenesis of various human diseases by causing apoptosis or cell dysfunction, which could lead to the activation of innate or adaptive immunity [10].

Alongside endogenous enzymes, multiple studies have discovered the existence of various exogenous factors, mostly found in foods (fruits and vegetables), such as vitamins or carotenoids that work as cofactors and assist endogenous enzymes in protecting our cells against oxidant stress by working on different signaling paths, cells chemotaxis, phagocytosis, etc. [11].

It is well established that certain substances contained in food can have powerful positive effects on human health in general, and specifically regarding the prevention and treatment of immune disorders; these molecules are named “Nutraceuticals” and, in recent decades, research has focused on understanding their clinical value and effectiveness [12,13,14,15].

In a previous review, we analyzed the potential effectiveness of nutraceuticals in autoimmune disorders [16]. This new article aims to describe the role of said nutraceuticals in treating allergies and related disorders, focusing on their ability to reduce oxidative stress and therefore modulate disease pathogenesis.

### 1.1. Exogenous Antioxidants

The antioxidant system represents a key mechanism in neutralizing oxidative processes. These molecules can be classified into two main groups: endogenous antioxidants, which are physiologically produced by the body, and exogenous antioxidants, which are obtained through diet or supplementation. Exogenous antioxidants encompass a wide spectrum of compounds, including vitamins, carotenoids, polyphenols, and minerals [17].

#### 1.1.1. Vitamins

Vitamin D, a pleiotropic hormone with well-known roles in calcium and phosphorus homeostasis, also exerts significant immunomodulatory functions [18,19]. It suppresses the activity of type 2 T helper cells (Th2) and inhibits B cell proliferation, thereby reducing immunoglobulin E (IgE) production [18].

Several observational studies have found a high prevalence of vitamin D deficiency among children with allergic disorders [19]. However, there is considerable evidence that vitamin D supplementation can safely alleviate the severity of atopic dermatitis and alleviate the symptoms of allergic rhinitis in the pediatric population [20].

Vitamin E is a lipid-soluble and potent antioxidant that primarily prevents lipid peroxidation. It has also been identified as an environmental factor capable of influencing early-life allergy development and modulating immune responses following allergen sensitization. The seemingly divergent outcomes associated with vitamin E are attributable to the distinct biological activities of its isoforms. Mechanistic studies have shown that α-tocopherol and γ-tocopherol exert opposing effects on allergic inflammation and disease progression: α-tocopherol reveals anti-inflammatory properties, whereas γ-tocopherol promotes pro-inflammatory responses in allergy and asthma [21,22].

Vitamin C is a small, water-soluble antioxidant derived from glucose, predominantly present in fruits and vegetables, particularly citrus species. Evidence from reviewed studies highlights its role in allergic and immunological disorders through anti-inflammatory, antioxidant, and immunomodulatory mechanisms [23]. In a murine model of allergic asthma, 24-day administration of vitamin C markedly reduced total white blood cell counts, the proportion of neutrophils and eosinophils, and peribronchial inflammatory cell infiltration. Treatment also decreased both the incidence and severity of moderate to severe asthma, as well as airway hyperresponsiveness to histamine [24]. investigations in patients with allergic rhinitis further demonstrated that vitamin C supplementation alleviated hallmark symptoms, including sneezing, lacrimation, pruritus, and malaise [25].

Vitamin A is a fat-soluble nutrient that is crucial for maintaining epithelial barriers and regulating immune responses. In atopic conditions, it supports skin and mucosal integrity and modulates T cell activity. Low levels of vitamin A have been linked to increased risk of atopic dermatitis and allergic airway inflammation, indicating its potential benefit in managing these diseases [26].

#### 1.1.2. Polyphenols

Polyphenols are a class of secondary metabolites synthesized by plants. Among the numerous polyphenols identified, approximately 8000 belong to the flavonoid family [27]. Flavonoids are hydroxylated polyphenolic compounds characterized by two or more aromatic rings linked via a heterocyclic pyran and at least one attached aromatic hydroxyl group. They are categorized according to structural differences into subgroups such as flavanones, flavones, isoflavones, flavonols, and anthocyanins [28].

Flavonoids have attracted interest as potential therapeutic agents due to their wide-ranging health benefits, including anti-inflammatory, antioxidant, and anti-allergic effects [29,30,31]. Various flavonoid sources have been investigated, either individually or in combination. Collectively, the evidence indicates that flavonoid supplementation may be beneficial in allergic conditions such as allergic rhinitis, asthma, and atopic dermatitis, without causing significant adverse effects [32].

Resveratrol is a natural polyphenol found in foods such as grapes, berries, and peanuts. It exhibits antioxidant, anti-inflammatory, and immunomodulatory effects [33].

Studies suggest that resveratrol may have protective roles in cardiovascular and neurodegenerative diseases, cancer, and inflammatory and allergic conditions [34]. Mechanistically, it can reduce oxidative stress, modulate key signaling pathways such as NF-κB and MAPK, and influence immune cell activity [35].

#### 1.1.3. Coenzyme Q10

Coenzyme Q10 (CoQ10) is a lipid-soluble, biologically active quinone consisting of a benzoquinone ring linked to an isoprenoid side chain. Beyond its primary role in mitochondrial bioenergetics, CoQ10 has been reported to have multiple functions, including involvement in ROS generation for cellular signaling, regulation of the cellular redox state, contribution to proton gradient formation in the endomembrane and plasma membrane, and modulation of membrane structure and phospholipid composition [36]. Nevertheless, its most prominent and clinically relevant function remains its antioxidant capacity. As an antioxidant, CoQ10 has shown potential as a therapeutic agent in diseases in which oxidative stress is a key pathological feature [37].

#### 1.1.4. Minerals

Some minerals are fundamental for the proper activity of the immune system [38].

They also contribute to the regulation of muscle contraction, nerve transmission, and the body’s water balance. At the same time, minerals act as structural components, particularly in bone formation, making them indispensable elements of human nutrition [39].

##### Zinc

The trace element zinc is indispensable for proper immune responses. Zinc deficiency is frequently associated with increased susceptibility to allergic disorders [40]. In an animal model of asthma, zinc deficiency was linked to heightened airway hyperresponsiveness compared to normal zinc intake, whereas zinc supplementation reduced inflammatory cell infiltration and improved clinical outcomes [41].

##### Selenium

Selenium is an essential trace element crucial for immune competence. Selenium deficiency impairs immune responses, while supplementation enhances immune function [42]. As a key component of glutathione peroxidase (GSH-Px), a major antioxidant enzyme, selenium helps reduce peroxides, protecting against inflammation-induced membrane damage and excessive oxidative stress [43]. Human studies have shown that lower serum selenium levels are associated with an increased risk of asthma [44]. Moreover, dietary selenium supplementation exhibits a synergistic anti-asthma effect when combined with α-tocopherol (vitamin E), the isoform with anti-inflammatory properties, attenuating airway inflammation and Th2-related cytokine production [45].

### 1.2. Probiotics

Probiotics are defined by the World Health Organization and the Food and Agriculture Organization of the United Nations as “live microorganisms which, when administered in adequate amounts, confer a health benefit on the host” [46]. Interest in the use of probiotics for the treatment of allergic disorders stems from their therapeutic potential, as in vitro studies have shown that they help reduce inflammatory cytokines and improve intestinal permeability [47]. These effects could be beneficial in managing allergies. Allergic disorders are characterized by an imbalance between Th1 and Th2 cytokines, with a predominance of the Th2 response. This imbalance leads to the activation of Th2 cytokines and the release of interleukin-4 (IL-4), IL-5, and IL-13, as well as stimulating IgE production. Probiotics may influence toll-like receptors and peptidoglycan recognition proteins in enterocytes, thereby promoting dendritic cell activation and a Th1-type immune response [48,49,50].

### 1.3. Medicals Plants

#### 1.3.1. L’Allium Sativum

Garlic is a spice with remarkable medicinal properties, known for its antibacterial, anticoagulant, and antioxidant effects [51]. Experimental studies have shown that garlic extract can significantly reduce allergic airway inflammation, as observed in murine models of allergic airway inflammation [52].

Specifically, research has demonstrated that the intraperitoneal administration of three doses of aged garlic extract led to a marked reduction in key indicators of allergic airway inflammation [52]. These included a decrease in the percentage of eosinophils in the bronchoalveolar lavage, the presence of eosinophils in peribronchial lung tissues, IgG levels in both lavage and serum, and a reduction in the number of mucus-secreting goblet cells and inflammation in peribronchial and perivascular regions [52].

#### 1.3.2. Capsaicin

*Capsicum annuum* L., a member of the Solanaceae family and classified within the Magnoliopsida class, is a herbaceous plant that may grow as either an annual or a short-lived perennial. It is widely valued for both its culinary uses and its medicinal properties. In traditional medicine systems—particularly in China—the fruit is used to warm the body, dispel sensations of internal cold, and aid digestion. It contains a range of bioactive compounds, most notably capsaicin, the main pungent agent, along with other capsaicinoids and carotenoid pigments. Scientific evidence has shown that capsaicin can effectively inhibit lipid peroxidation in the membranes of red blood cells, liver tissue, and mitochondria in animal models [53]. Furthermore, it can prevent the oxidative degradation of low-density lipoproteins (LDL) in humans [54].

In certain scenarios, its antioxidant efficacy has been found to exceed that of vitamin E [55].

Dietary intake of capsaicin may reduce oxidative stress and enhance cellular antioxidant defense systems by preventing ROS from depleting glutathione stores. Capsaicin has also been observed to counteract the suppressive effects of high blood cholesterol on key antioxidant enzymes such as glutathione reductase, glutathione S-transferase, and superoxide dismutase [56].

Additionally, it has the capacity to neutralize free radicals, including 1,1′-diphenyl-2-picrylhydrazyl (DPPH) [53,56].

#### 1.3.3. Curcumin

Curcumin is widely recognized for its diverse pharmacological properties, which include antioxidant, anti-inflammatory, antimicrobial, antiviral, antifungal, and antitumor activities [57]. These characteristics make it a promising candidate for both monotherapy and combination therapy (adjuvant therapy) in the management of various diseases [58].

Given the pivotal role of mast cells in allergic reactions and immediate hypersensitivity responses, the impact of curcumin on these immune cells has been explored [59].

Research has demonstrated that curcumin significantly inhibits the degranulation of stimulated rat peritoneal mast cells (RPMCs) in in vitro models [60]. This inhibition appears to be dose-dependent and is likely mediated by a reduction in intracellular Ca^2+^ levels, which play a crucial role in histamine release. Since the binding of IgE to its high-affinity receptor (FcεRI) is a key event in the activation of mast cells and basophils, leading to allergic responses, curcumin’s ability to interfere with this receptor could suppress downstream signaling pathways [61]. This, in turn, may prevent basophil activation and degranulation, ultimately reducing the release of pro-inflammatory cytokines, histamine, and lipid-derived mediators [62]. The immunomodulatory and anti-allergic potential of curcumin has been further confirmed through both in vitro and in vivo studies. Notably, bisdemethoxycurcumin (BDMC), a naturally occurring curcuminoid, has been shown to effectively inhibit the release of β-hexosaminidase and histamine, which correlates with the clinical alleviation of allergic rhinitis symptoms [61,63].

### 1.4. Melatonin

Melatonin is a hormone secreted by the pineal gland in accordance with the circadian rhythm. Its antioxidant and anti-inflammatory properties play a key role in supporting physiological functions and maintaining homeostasis [64].

Emerging evidence suggests that melatonin may also influence the pathogenesis of allergic and atopic diseases, potentially modulating immune responses and reducing symptom severity in conditions such as atopic dermatitis and allergic rhinitis [64,65].

### 1.5. L-Arginine

L-arginine is classified as a semi-essential or conditionally essential amino acid, since its availability depends not only on dietary intake but also on endogenous synthesis. The latter occurs through protein turnover and the conversion of L-citrulline in a two-step process catalyzed by argininosuccinate synthase (ASS) and argininosuccinate lyase (ASL), which requires cooperation between intestinal mucosa and renal proximal tubule cells [66]. Alterations in L-arginine homeostasis have direct implications for the respiratory system and, in allergic asthma, have been linked to airway hyperresponsiveness and the structural remodeling of the airway wall [67]. This is largely explained by the fact that the amount of L-arginine available to nitric oxide synthase (NOS) regulates the production of nitric oxide (NO), an endogenous bronchodilator that is crucial for airway function [67]. Thus, beyond its role in protein synthesis, L-arginine exerts a broader metabolic function by serving as a substrate for multiple enzymes, among which NOS is one of the most relevant [68].

### 1.6. Omega-3

Several preclinical studies have highlighted the role of ω-3 Polyunsaturated Fatty Acids (PUFAs) as molecules with protective effects against allergic inflammation [69].

Notably, docosahexaenoic acid appears to contribute to protection against allergic diseases by reducing IgE production by human B cells, inhibiting Th2 cell polarization, and promoting IL-10 production, which leads to an increased number of dendritic cells (DCs) [70,71]. The beneficial effects of ω-3 PUFAs are not limited to their ability to counteract antigen presentation to naïve T cells, but also include a reduction in dendritic cell immunogenicity [69]. In vitro studies conducted by Zeyda et al. have shown that treating monocyte-derived DCs with PUFAs can interfere with their immunogenic function, leading to altered surface molecule expression and a decrease in cytokine release [69].

## 2. Methods

We searched PubMed and Embase for articles published from March 2015 to April 2025 using the following keywords: ‘allergic disease, oxidative stress, reactive oxygen species, exogenous antioxidants, antioxidant supplementation, treatment, nutraceuticals’. Only articles published in English were considered. The articles were then sorted by relevance and screened for consistency with the aim of the review, with articles that included the effects of nutraceuticals on allergic disease also being selected. Although our main objective was to focus on results from the last 10 years, we could not ignore some of the most relevant research papers from prior to the last decade.

## 3. Results and Discussion

Table 1 summarizes antioxidants’ mechanism of action and their impact on clinical outcomes in allergic diseases. We describe the literature results for each disease in the following paragraphs.

### 3.1. Bronchial Asthma

Bronchial asthma (BA) is a chronic disease caused by inflammation and reversible bronchoconstriction [86]. Although asthma’s pathogenesis is not fully understood, it is well known that ROS overproduction in asthma patients is associated with increased airway inflammation [72]. The first line of treatment for asthma mainly comprises anti-inflammatory agents (especially corticosteroids) and bronchodilators; however, long-term therapy with corticosteroids leads to steroid resistance and systemic side effects (weight gain, osteoporosis, etc.), which leads us to analyze further therapeutic strategies [73].

Multiple studies and clinical evidence have suggested the role of antioxidant agents, especially nutraceuticals, as an adjunctive therapy for asthmatic patients [87].

A meta-analysis published by Nutrition Hospitalaria in 2023 evaluated the possible clinical relevance of the administration of probiotics in asthmatic patients. They included ten randomized controlled studies, and a total of 1101 people were investigated. They proved that lung inflammation (evaluated by measuring fractional exhaled nitric oxide (FeNO) levels), asthma symptom severity and number of acute episodes of asthma improved in patients treated with probiotics compared to the control group [88].

Another study, conducted in Japan in 2015, analyzed the effects of vitamin E in asthmatic patients and showed that gamma tocotrienol was able to reduce airway smooth muscle cells’ proliferation and migration, and therefore has therapeutic relevance regarding airway remodeling [89].

Another study, conducted in Romania and published in 2021, demonstrated that vitamin D supplementation reduced oxidative forces and increased the antioxidant capacity in a mouse model of ovalbumin-induced acute asthmatic airway inflammation [90]. While promising, it should be acknowledged that these results refer to mouse cells and do not automatically translate to therapeutic effect in humans; however, many human clinical trials have analyzed and confirmed the clinical value of vitamin D supplementation in asthmatic patients [91,92,93].

For example, this 12-week double-blind randomized control trial demonstrated that there is a significant difference in terms of FEV1/FVC ratio in patients administered a dietary supplementation with 125 μg/d vitamin D compared to the placebo group. In this study, an association between plasma vitamin D concentration and IL-10 and TNF-*α* concentrations was also highlighted [91].

Many studies have evaluated the role of selenium in bronchial asthma [92,93].

This study showed how there is a fine correlation between selenium intake and lung function: they included 4541 patients, evaluated lung function in terms of FEV1/FVC and divided selenium intakes into crescent levels. They noted that higher selenium intakes improved lung function based on an increase in both FEV1 and FVC and therefore suggest that patients who suffers from BA consume 137–200 µg of selenium daily [93].

As mentioned before, minerals have been proven to be powerful antioxidants given their ability to modulate cells’ response, enzymes’ function and production, etc.; this study, for example, particularly enhanced the benefits of zinc supplementation in pediatric patients with bronchial asthma in terms of lung inflammation and clinical symptoms [94].

### 3.2. Atopic Dermatitis

Atopic dermatitis (AD) is a chronic, relapsing inflammatory skin disorder with a multifactorial etiology [95]. The prevalence of AD ranges from 10–20% in childhood to 2–8% in adulthood, with primary clinical manifestations including pruritus and eczematous lesions, particularly affecting the flexural regions [96]. Current treatment strategies for AD encompass topical and systemic corticosteroids, immunosuppressive agents, and biologic therapies such as monoclonal antibodies and Janus kinase (JAK) inhibitors [96].

The pathophysiology of AD is complex, involving a combination of genetic predisposition, impaired epidermal barrier function, and an imbalanced inflammatory response to environmental triggers [95].

ROS play a central role in the pathogenesis of AD, as they react with macromolecules—including lipids, proteins, nucleic acids, and carbohydrates—initiating chain reactions that lead to cellular damage and death. ROS also induce the upregulation of genes encoding pro-inflammatory cytokines, thereby perpetuating a self-amplifying inflammatory cycle [74].

Clinical evidence suggests that antioxidant agents may serve as adjunctive therapies in AD management and prevention, potentially reducing reliance on corticosteroids and mitigating their associated adverse effects [97]. Several antioxidant molecules, including vitamins D, E, A, and melatonin, have been evaluated. Vitamin D appears particularly promising, with one randomized controlled trial (RCT) involving 60 patients reporting significant improvement following administration of 1600 IU/day for 60 days [98].

Data on vitamin E are limited, with only a single RCT indicating potential benefit [99]. Similarly, melatonin administered at 3–6 mg/day has been shown in randomized trials to significantly reduce SCORAD scores, a validated measure of atopic dermatitis severity that evaluates lesion extent, intensity, and patient-reported symptoms such as itch and sleep loss [100].

Numerous flavonoid extracts have been investigated for their therapeutic potential in AD. Compounds such as quercetin and kaempferol suppress IL-4, IL-5, and IL-13 secretion, eosinophil recruitment, and mast cell activation, key hallmarks of AD [31].

Quercetin, in particular, exerts multifaceted effects by modulating oxidative stress, inflammatory pathways, cytokine production, and mast cell activity, making it a promising natural therapeutic agent [101]. It regulates pathways including NF-κB and MAPK and influences mast cell and histamine responses, supporting its potential use in anti-dermatitis formulations [102].

Epigallocatechin gallate (EGCG), a predominant flavone-3-ol polyphenol found in green tea leaves, has demonstrated anti-inflammatory, antioxidant, and immunomodulatory properties relevant to AD management [103]. Topical applications of EGCG have been reported to significantly reduce skin inflammation, ear thickness, and immune cell infiltration [31].

A 2021 meta-analysis by Sun et al. examined the effects of mixed lactobacillus and bifidobacterium strains on AD in infants under three years old, analyzing nine double-blind, placebo-controlled RCTs encompassing 2093 infants [104]. Maternal and/or infant supplementation with mixed probiotic strains effectively reduced eczema incidence. Subgroup analysis showed that the probiotic mixture was preventive in infants both with and without a family history of atopic disease, with prenatal administration demonstrating greater efficacy than postnatal intervention alone. Daily probiotic doses ≤1 × 10^9^ and >1 × 10^9^ CFU were both effective in reducing AD incidence [75].

Prenatal dietary and environmental exposures have been associated with the development of chronic diseases, with maternal PUFA status representing a modifiable factor influencing AD risk [76]. Higher prenatal n-6 PUFA levels were linked to increased odds of childhood AD at 4–6 years, particularly in children of mothers with a history of atopic disease [105].

### 3.3. Allergic Rhinitis

Allergic rhinitis is a very common disorder affecting between 10% and 30% of the population [77,78]. From a pathophysiological perspective, it is triggered by exposure to allergens, leading to an IgE-mediated inflammatory response at the nasal mucosa [77,78].

This process results in sensitization to these allergens and the subsequent onset of symptoms, including rhinorrhea, itching, and nasal congestion [77,78]. These symptoms can significantly impact patients’ quality of life and generate substantial economic burden.

In this context, probiotics have emerged as a potential therapeutic option for allergic rhinitis, attracting increasing attention within the scientific community [106].

Recent reviews have shown that probiotics provide benefits in the management of allergic rhinitis, improving patients’ quality of life and reducing the need for medication [107,108].

The mechanisms through which probiotics may modulate atopic diseases remain unclear. Evidence from murine models suggests that probiotics may promote type 1 T helper (Th1) cell-mediated immunity by suppressing type 2 T helper (Th2) responses [109].

Other studies have shown that probiotics may enhance the presence of regulatory T cells (Treg) through interactions with the gut microbiota [106,107,108,109,110].

### 3.4. Urticaria and Angioedema

Urticaria and angioedema are two common and linked disorders those clinical manifestations are, respectively, the presence of wheals (areas of pruritic skin edema) and localized areas of soft-tissue swelling (as proof of the involvement of the deeper dermis and subcutaneous tissue in angioedema) [79,80]. The pathogenesis is mostly due to mast cell (MC) degranulation and the release of pro-inflammatory mediators, such as histamine, which causes the classic clinical manifestation, and, notably, urticaria, is classified as acute and chronic using a six-week timeline [79].

ROS have been proven to play an important, although not fully understood, role in the pathogenesis of chronic urticaria and angioedema; therefore, the research focused on analyzing any possible clinical relevance of various exogenous antioxidants regarding chronic urticaria (CU) [81].

A recent meta-analysis, published in 2021 by the *International Journal of Environmental Research and Public Health,* showed that chronic urticaria is usually linked to low vitamin D levels and supplementation of said vitamins positively affected clinical outcome in terms of quality-of-life scores [111].

As mentioned before, urticaria’s pathogenesis mostly comprises mast cell degranulation, which is influenced by oxidative stress. Many studies have therefore investigated the potential role of exogenous antioxidants in reducing mast cell activation [112].

For example, multiple studies have analyzed the potential ability of various polyphenols (including quercetin and resveratrol) in regulating MC degranulation and the synthesis of arachidonic acid metabolites [113,114,115,116].

These studies highlighted a global reduction in interleukin production, the expression of cytokines and pro-inflammatory molecular pathways that lead to an inhibition of MC degranulation and the synthesis of arachidonic acid metabolites, which opens the door to a potential clinical relevance of said nutraceuticals in patients affected by mast cell-related diseases, including CU [113,114,115,116].

Recently, probiotics have been studied for their antioxidant properties. This study analyses the potential clinical relevance of probiotics as antioxidants in patients affected by CU: a total of 38 patients were enrolled, divided into a control group and intervention group; these groups were administered an antihistamine and placebo and antihistamine and probiotics, respectively. The clinical outcome was measured by urticaria activity score over 7 days (UAS7), and a more significant score reduction was observed in patients treated with probiotics [117].

### 3.5. Allergic Conjunctivitis

Allergic conjunctivitis (AC) is triggered by environmental allergens and manifests with ocular symptoms such as itching and excessive tearing [118]. Traditional pharmacological treatments, however, present some limitations: the clinical effect of a single antihistamine dose usually lasts only 24–36 h, after which symptoms recur, while intranasal corticosteroids, once discontinued, allow for inflammation and symptoms to reappear within just a few days [119,120]. Moreover, in highly allergic patients, during intense allergen exposure or in the presence of comorbidities, these therapies may not fully suppress the allergic response, highlighting the potential value of additional treatments [121]. In this context, a new oral nutraceutical has been developed for rhinoconjunctivitis. The formulation combines Perilla frutescens (80 mg, dry extract), Quercetin (150 mg), and vitamin D3 (5 mcg, 200 IU). Seeds of Perilla frutescens provide high levels of rosmarinic acid and flavonoids such as luteolin, apigenin, and chrysoeriol, molecules with well-documented anti-allergic properties demonstrated in both in vitro and in vivo models [122]. Clinical evidence supports the efficacy of this nutraceutical. An open study in adults with seasonal rhinoconjunctivitis showed a significant reduction in symptoms and in the use of anti-allergic medications [123]. Furthermore, the beneficial effect persisted during the later phases of treatment: between the third and fourth week, when some patients experienced a return of symptoms despite drug therapy, the supplement appeared to maintain control [121]. When used as an adjunct, the oral combination of Perilla frutescens, quercetin, and vitamin D3 enhanced the effects of standard treatments, leading to fewer relapses during therapy in children with rhinoconjunctivitis [121]. Given its favorable safety profile, it can be taken for prolonged periods, for example, throughout the pollen season in patients allergic to pollens, or during autumn and winter in those sensitized to mites [121].

### 3.6. Food Allergies

In the field of food allergy, as in other allergic disorders, probiotics have been shown to positively modulate both the gut microbiota and the immune system [83,124].

Preclinical and clinical studies conducted in recent years have demonstrated a promising role of probiotics in the prevention and treatment of food allergy [124].

Among these, one study demonstrated that *Lactobacillus plantarum* HM22 increased serum levels of tolerance-promoting cytokines such as IL-10, IFN-γ, and TGF-β, while decreasing total IgE and IL-4 levels, in mice with α-lactalbumin-induced allergy [82].

Other studies showed that treatment with *Lactobacillus acidophilus* KLDS 1.0738 can suppress the TLR4/NF-κB signaling pathway through the modulation of microRNA miR-146a, thereby reducing the downstream production of inflammatory factors [83].

The strain that demonstrated the greatest overall efficacy was *Bacillus coagulans* 09.712, through the improvement in epithelial barrier function and the increase in lymphocyte proliferation. Moreover, this strain was shown to enhance the production of CD4+Foxp3+ regulatory T cells, leading to the suppression of the pro-inflammatory Th17 response in this murine model [84,85].

Other studies highlighted that quercetin protects low-density lipoproteins from damage, acting as a potent antioxidant by reducing free radicals.

Although capsaicin supplementation does not reduce the main features of food allergy, such as IgE production and body weight loss, it exerts beneficial effects on several related parameters. Specifically, a decrease in macrophage infiltration and lower IL-33 expression in the proximal jejunum have been reported, indicating a reduction in local inflammatory responses [125]. Capsaicin has also been shown to reduce hepatic triglyceride accumulation and intestinal hydroperoxide levels. Overall, these findings suggest that oral supplementation with capsaicin may attenuate the inflammation and oxidative stress associated with food allergy, potentially contributing to an improved prognosis and disease progression [125].

## 4. Discussion

**Figure 1:**
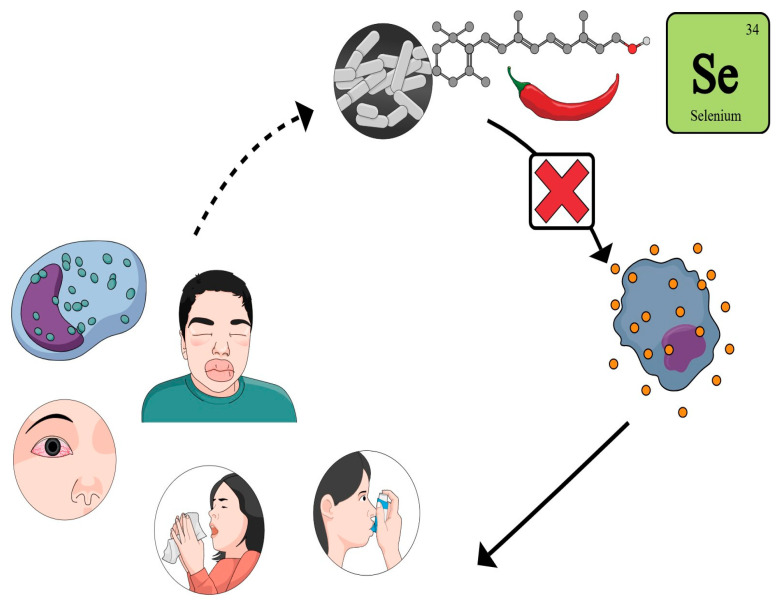
Graphical representation and summary of the role of nutraceuticals, including capsaicin, selenium, and probiotics, in modulating oxidative stress and immune responses in allergic diseases. These compounds contribute to the reduction in reactive oxygen species (ROS) and consequently improve symptoms and quality of life in patients with allergic rhinitis, allergic conjunctivitis, asthma, and angioedema.

The analyzed studies highlight the key role of oxidative stress in the pathogenesis and progression of allergic diseases (Figure 1). To counteract the effects of various oxidizing agents in the onset of these conditions, both exogenous and endogenous antioxidant substances have been evaluated. Specifically, the effectiveness of different nutraceuticals has been investigated for each allergic disease.

Several studies demonstrated the effectiveness of nutraceuticals in the treatment of allergic bronchial asthma [87,88,91,92,94].

These studies have shown that the use of probiotics in patients with bronchial asthma can reduce airway inflammation, symptom severity, and the frequency of asthma exacerbation [88].

Moreover, supplementation with vitamin D and vitamin E appears to enhance the antioxidant response in patients with bronchial asthma [89,90]. The integration of minerals such as zinc and selenium has also proven effective in improving respiratory function [92,94].

The use of nutraceuticals has also been shown to be crucial in the treatment of atopic dermatitis. The effectiveness of various antioxidant substances—such as vitamin A, vitamin E, vitamin D, and melatonin—was evaluated in this context [98,99,100]. Probiotics also exert immunomodulatory effects on atopic dermatitis, balancing the Th1/Th2 immune response, stimulating Th1, and decreasing Th2 response through the secretion of different cytokines [75,76,104].

In this context, it is worth noting that other natural compounds have also shown therapeutic potential in atopic dermatitis. Specifically, quercetin exerts multifaceted effects by modulating oxidative stress, inflammatory pathways, cytokine production, and mast cell activity, thus emerging as a promising candidate for controlling cutaneous inflammatory processes [101]. Similarly, topical applications of EGCG have been reported to significantly reduce skin inflammation, ear thickness, and immune cell infiltration [31].

Moreover, maternal PUFA status has been identified as a modifiable factor influencing AD risk, with higher prenatal n-6 PUFA levels being associated with an increased likelihood of childhood AD, especially in the offspring of atopic mothers [76,105].

Recent reviews have suggested that probiotics also may have significant beneficial effects on the management of allergic rhinitis, with the potential to improve patients’ quality of life and reduce medication use [106,107,108,109,110].

In the treatment of urticaria, the use of polyphenols (including quercetin and resveratrol) may help regulate mast cell degranulation [113,114,115,116]. Additionally, supplementation with vitamin D and probiotics was shown to improve symptoms in patients with chronic urticaria and enhance their quality of life [111].

A recent oral nutraceutical formulation for allergic rhinoconjunctivitis, containing *Perilla frutescens*, quercetin, and vitamin D3, has been investigated in adults with seasonal rhinoconjunctivitis. The study reported a significant reduction in symptom severity as well as in the use of concomitant antiallergic medications [121].

Probiotics have shown a promising role in food allergies [83,124]. *Lactobacillus plantarum* HM22 enhances IL-10, IFN-γ, and TGF-β, while reducing IgE and IL-4 [82], whereas *Lactobacillus acidophilus* KLDS 1.0738 modulates the ILR4/NF-κB pathway via miR-146a, lowering inflammatory mediators. The most effective strain, *Bacillus coagulans* 09.712, improves epithelial barrier function, promotes CD4+Foxp3+ Treg expansion, and suppresses the Th17 response [84].

In addition to probiotics, natural compounds also contribute: quercetin protects LDL against oxidative damage and capsaicin enhances antioxidant defenses by preserving glutathione and preventing the cholesterol-induced inhibition of key enzymes.

## 5. Perspectives

In this review, we explored a substantial body of evidence that highlights the complex and significant relationship between reactive oxygen species (ROS), antioxidants, and allergic diseases. Oxidative stress, while a natural and often necessary biochemical process within the body, can become harmful when unregulated. Under normal physiological conditions, various endogenous enzymatic systems—such as superoxide dismutase (SOD), catalase (CAT), and glutathione peroxidase (GPx)—play a crucial role in maintaining cellular homeostasis by neutralizing excess ROS and protecting cells from oxidative damage.

However, when cells are exposed to a variety of internal and external stressors—such as environmental pollutants, allergens, infections, or metabolic disturbances—these endogenous antioxidant defenses may become overwhelmed. In such situations, the balance between oxidants and antioxidants is disrupted, leading to a state of oxidative stress that can contribute to inflammation, cellular dysfunction, and tissue damage. To counteract this imbalance, exogenous antioxidants—primarily obtained through diet or supplementation—can serve as valuable allies in supporting the body’s defense mechanisms and restoring redox homeostasis [126].

The ability of ROS to damage cellular components, including DNA, proteins, and lipids, plays a key role in the pathophysiology of several allergic conditions. This oxidative damage not only affects immune cell function but may also exacerbate inflammatory responses and compromise the integrity of epithelial barriers, thereby promoting the onset and progression of allergic diseases. Although the link between the proposed antioxidant action of a nutraceutical and the specific clinical improvements is sometimes assumed rather than explicitly reported, numerous studies have demonstrated that dietary supplementation with exogenous antioxidants can yield beneficial effects in managing allergies, helping to reduce symptom severity and improve overall clinical outcomes.

## 6. Conclusions

In conclusion, while the current evidence underscores the importance of antioxidants in modulating oxidative stress and its implications for allergic disorders, further well-designed clinical trials and mechanistic studies are needed. These future investigations should aim to better elucidate the specific roles of different types of antioxidants, their interactions, and optimal therapeutic strategies for the prevention and treatment of allergies and related conditions.

## Figures and Tables

**Table 1:** The role of antioxidants in allergic diseases: mechanisms of action and impact on clinical outcomes.

Allergic Disease	Antioxidants	Outcomes
ASTHMA	Probiotics Vit D, Vit E Selenium, Zinc	Probiotics (10 RCTs, *n* = 1101) significantly reduced FeNO levels, asthma symptom severity, and frequency of acute exacerbations. Vitamin D supplementation (125 μg/d, 12 weeks) improved FEV1/FVC ratio and modulated cytokine profile (↑IL-10, ↓TNF-α). Selenium intake (137–200 μg/d) correlated with improved lung function (↑FEV1, ↑FVC) in a cohort of 4541 patients. Vitamin E (γ-tocotrienol) reduced airway smooth muscle hyperplasia in preclinical models. Zinc supplementation showed benefits on lung inflammation in pediatric asthma [72,73].
ATOPIC DERMATITIS	Vit D Vit E Vit A Melatonin EGCG Probiotics PUFA Quercetin	In atopic dermatitis, antioxidants and nutraceuticals are emerging as promising supportive therapies. Vitamins such as D, E, A, and melatonin may help decrease clinical severity, improving itch, skin lesions, and sleep quality. Polyphenols like quercetin and EGCG modulate oxidative stress and key inflammatory pathways, contributing to better immune balance. Probiotics have been shown to lower the incidence of eczema, particularly when administered prenatally, while an appropriate maternal n-3/n-6 PUFA ratio during pregnancy appears protective against AD. Overall, these interventions may lessen corticosteroid dependence and enhance long-term disease management [74,75,76].
ALLERGIC RHINITIS	Probiotics	Probiotics, part of nutraceuticals, have shown promising results in allergic rhinitis, such as decreased symptoms and reduced medication use. Their great importance lies in modulating the immune response (shifting Th2 dominance, enhancing Treg activity, and altering the gut microbiota), suggesting a supportive role in managing allergic inflammation and improving quality of life [77,78].
URTICARIA AND ANGIOEDEMA	Vit D	In urticaria and angioedema, mast cell degranulation with histamine release is the main pathogenic mechanism, while ROS contribute to chronic inflammation. Evidence shows that low vitamin D levels are often associated with chronic urticaria, and supplementation leads to improved symptom control and quality of life. Correcting vitamin D deficiency may therefore represent a useful adjunctive strategy in the management of these conditions [79,80,81].
FOOD ALLERGY	Quercetin Capsaicin Probiotics	Quercetin protects LDL from oxidative damage through its antioxidant activity, while capsaicin reduces oxidative stress, preserves glutathione, and enhances key antioxidant enzymes. Probiotics show promising immunomodulatory effects in food allergies: *Lactobacillus plantarum* HM22 increases regulatory cytokines and reduces IgE and IL-4, *Lactobacillus acidophilus* KLDS 1.0738 suppresses the TLR4/NF-κB pathway via miR-146a, and *Bacillus coagulans* 09.712 strengthens the epithelial barrier, stimulates Treg cells, and decreases Th17 responses. These findings suggest that bioactive compounds and probiotics may significantly modulate oxidative stress and immune responses, representing promising strategies for the prevention and treatment of food allergies [82,83,84,85].

↑: Augmented; ↓: reduced.

## Data Availability

Not applicable.

## Abbreviations

DPPH	1,1′-diphenyl-2-picrylhydrazyl
CAT	Catalase
AC	Allergic conjunctivitis
AR	Allergic rhinitis
AOX	Antioxidant processes
ASL	Argininosuccinate lyase
ASS	Argininosuccinate synthase
AD	Atopic dermatitis
BDMC	Bisdemethoxycurcumin
BA	Bronchial asthma
CoQ10	Coenzyme Q10
CU	Chronic urticaria
DCs	Dendritic cells
EGCG	Epigallocatechin gallate
FeNO	Fractional exhaled nitric oxide
GPx	Glutathione peroxidase
HOCI	Hypochlorous acid
LDL	Low-density lipoproteins
MC	Mast cells
NO	Nitric oxide
NOS	Nitric oxide synthase
PUFAs	Polyunsaturated fatty acids
ROS	Reactive oxygen species
SOD	Superoxide dismutase
UAS7	Urticaria activity score over 7 days

## References

[1] Thannickal V.J., Fanburg B.L. (2000). Reactive Oxygen Species in Cell Signaling. Am. J. Physiol.-Lung Cell. Mol. Physiol..

[2] D’Autréaux B., Toledano M.B. (2007). ROS as Signalling Molecules: Mechanisms That Generate Specificity in ROS Homeostasis. Nat. Rev. Mol. Cell Biol..

[3] Brieger K., Schiavone S., Miller F.J., Krause K.-H. (2012). Reactive Oxygen Species: From Health to Disease. Swiss Med. Wkly..

[4] Yu Y., Cui Y., Niedernhofer L.J., Wang Y. (2016). Occurrence, Biological Consequences, and Human Health Relevance of Oxidative Stress-Induced DNA Damage. Chem. Res. Toxicol..

[5] Demple B., Amábile-Cuevas C.F. (1991). Redox Redux: The Control of Oxidative Stress Responses. Cell.

[6] Baker A., Lin C.-C., Lett C., Karpinska B., Wright M.H., Foyer C.H. (2023). Catalase: A Critical Node in the Regulation of Cell Fate. Free Radic. Biol. Med..

[7] Handy D.E., Loscalzo J. (2022). The Role of Glutathione Peroxidase-1 in Health and Disease. Free Radic. Biol. Med..

[8] Ortiz-Cerda T., Xie K., Mojadadi A., Witting P.K. (2023). Myeloperoxidase in Health and Disease. Int. J. Mol. Sci..

[9] Demirci-Çekiç S., Özkan G., Avan A.N., Uzunboy S., Çapanoğlu E., Apak R. (2022). Biomarkers of Oxidative Stress and Antioxidant Defense. J. Pharm. Biomed. Anal..

[10] Cross C.E., Halliwell B., Borish E.T., Pryor W.A., Ames B.N., Saul R.L., McCORD J.M., Harman D. (1987). Oxygen Radicals and Human Disease. Ann. Intern. Med..

[11] Carr A., Maggini S. (2017). Vitamin C and Immune Function. Nutrients.

[12] Martel J., Ojcius D.M., Ko Y.-F., Ke P.-Y., Wu C.-Y., Peng H.-H., Young J.D. (2019). Hormetic Effects of Phytochemicals on Health and Longevity. Trends Endocrinol. Metab..

[13] Aronson J.K. (2017). Defining ‘Nutraceuticals’: Neither Nutritious nor Pharmaceutical. Br. J. Clin. Pharmacol..

[14] Chandra S., Saklani S., Kumar P., Kim B., Coutinho H.D.M. (2022). Nutraceuticals: Pharmacologically Active Potent Dietary Supplements. BioMed Res. Int..

[15] Schmitt J., Ferro A. (2013). Nutraceuticals: Is There Good Science behind the Hype?. Br. J. Clin. Pharmacol..

[16] Mannucci C., Casciaro M., Sorbara E.E., Calapai F., Di Salvo E., Pioggia G., Navarra M., Calapai G., Gangemi S. (2021). Nutraceuticals against Oxidative Stress in Autoimmune Disorders. Antioxidants.

[17] Korczowska-Łącka I., Słowikowski B., Piekut T., Hurła M., Banaszek N., Szymanowicz O., Jagodziński P.P., Kozubski W., Permoda-Pachuta A., Dorszewska J. (2023). Disorders of Endogenous and Exogenous Antioxidants in Neurological Diseases. Antioxidants.

[18] Vasiliou J.E., Lui S., Walker S.A., Chohan V., Xystrakis E., Bush A., Hawrylowicz C.M., Saglani S., Lloyd C.M. (2014). Vitamin D Deficiency Induces TH2 Skewing and Eosinophilia in Neonatal Allergic Airways Disease. Allergy.

[19] Kuruvilla M.E., Lee F.E.-H., Lee G.B. (2019). Understanding Asthma Phenotypes, Endotypes, and Mechanisms of Disease. Clin. Rev. Allergy Immunol..

[20] Li Q., Zhou Q., Zhang G., Tian X., Li Y., Wang Z., Zhao Y., Chen Y., Luo Z. (2022). Vitamin D Supplementation and Allergic Diseases during Childhood: A Systematic Review and Meta-Analysis. Nutrients.

[21] Cook-Mills J.M., Averill S.H., Lajiness J.D. (2022). Asthma, Allergy and Vitamin E: Current and Future Perspectives. Free Radic. Biol. Med..

[22] Gęgotek A., Skrzydlewska E. (2023). Ascorbic Acid as Antioxidant. Vitamins and Hormones.

[23] Du Y.-T., Long Y., Tang W., Liu X.-F., Dai F., Zhou B. (2022). Prooxidative Inhibition against NF-κB-Mediated Inflammation by Pharmacological Vitamin C. Free Radic. Biol. Med..

[24] Kianian F., Karimian S.M., Kadkhodaee M., Takzaree N., Seifi B., Adeli S., Harati E., Sadeghipour H.R. (2019). Combination of Ascorbic Acid and Calcitriol Attenuates Chronic Asthma Disease by Reductions in Oxidative Stress and Inflammation. Respir. Physiol. Neurobiol..

[25] Ghalibaf M.H.E., Kianian F., Beigoli S., Behrouz S., Marefati N., Boskabady M., Boskabady M.H. (2023). The Effects of Vitamin C on Respiratory, Allergic and Immunological Diseases: An Experimental and Clinical-Based Review. Inflammopharmacol.

[26] Joshi M., Hiremath P., John J., Ranadive N., Nandakumar K., Mudgal J. (2023). Modulatory Role of Vitamins A, B3, C, D, and E on Skin Health, Immunity, Microbiome, and Diseases. Pharmacol. Rep..

[27] Serafini M., Peluso I., Raguzzini A. (2010). Flavonoids as Anti-Inflammatory Agents. Proc. Nutr. Soc..

[28] Al-Khayri J.M., Sahana G.R., Nagella P., Joseph B.V., Alessa F.M., Al-Mssallem M.Q. (2022). Flavonoids as Potential Anti-Inflammatory Molecules: A Review. Molecules.

[29] Tunon M., Garcia-Mediavilla M., Sanchez-Campos S., Gonzalez-Gallego J. (2009). Potential of Flavonoids as Anti-Inflammatory Agents: Modulation of Pro-Inflammatory Gene Expression and Signal Transduction Pathways. Curr. Drug Metab..

[30] Ullah A., Munir S., Badshah S.L., Khan N., Ghani L., Poulson B.G., Emwas A.-H., Jaremko M. (2020). Important Flavonoids and Their Role as a Therapeutic Agent. Molecules.

[31] Zawawi N.A., Ahmad H., Madatheri R., Fadilah N.I.M., Maarof M., Fauzi M.B. (2025). Flavonoids as Natural Anti-Inflammatory Agents in the Atopic Dermatitis Treatment. Pharmaceutics.

[32] De Almeida Brasiel P.G., Guimarães F.V., Rodrigues P.M., Bou-Habib D.C., Carvalho V.D.F. (2022). Therapeutic Efficacy of Flavonoids in Allergies: A Systematic Review of Randomized Controlled Trials. J. Immunol. Res..

[33] Harikumar K.B., Aggarwal B.B. (2008). Resveratrol: A Multitargeted Agent for Age-Associated Chronic Diseases. Cell Cycle.

[34] Kodali M., Parihar V.K., Hattiangady B., Mishra V., Shuai B., Shetty A.K. (2015). Resveratrol Prevents Age-Related Memory and Mood Dysfunction with Increased Hippocampal Neurogenesis and Microvasculature and Reduced Glial Activation. Sci. Rep..

[35] Zhang L.-X., Li C.-X., Kakar M.U., Khan M.S., Wu P.-F., Amir R.M., Dai D.-F., Naveed M., Li Q.-Y., Saeed M. (2021). Resveratrol (RV): A Pharmacological Review and Call for Further Research. Biomed. Pharmacother..

[36] Littarru G.P., Tiano L. (2007). Bioenergetic and Antioxidant Properties of Coenzyme Q10: Recent Developments. Mol. Biotechnol..

[37] Gutierrez-Mariscal F.M., Arenas-de Larriva A.P., Limia-Perez L., Romero-Cabrera J.L., Yubero-Serrano E.M., López-Miranda J. (2020). Coenzyme Q10 Supplementation for the Reduction of Oxidative Stress: Clinical Implications in the Treatment of Chronic Diseases. Int. J. Mol. Sci..

[38] Weyh C., Krüger K., Peeling P., Castell L. (2022). The Role of Minerals in the Optimal Functioning of the Immune System. Nutrients.

[39] Couce M.L., Saenz de Pipaon M. (2021). Bone Mineralization and Calcium Phosphorus Metabolism. Nutrients.

[40] Peroni D.G., Hufnagl K., Comberiati P., Roth-Walter F. (2023). Lack of Iron, Zinc, and Vitamins as a Contributor to the Etiology of Atopic Diseases. Front. Nutr..

[41] Faghfouri A.H., Zarezadeh M., Aghapour B., Izadi A., Rostamkhani H., Majnouni A., Abu-Zaid A., Kord Varkaneh H., Ghoreishi Z., Ostadrahimi A. (2021). Clinical Efficacy of Zinc Supplementation in Improving Antioxidant Defense System: A Comprehensive Systematic Review and Time-Response Meta-Analysis of Controlled Clinical Trials. Eur. J. Pharmacol..

[42] Bjørklund G., Shanaida M., Lysiuk R., Antonyak H., Klishch I., Shanaida V., Peana M. (2022). Selenium: An Antioxidant with a Critical Role in Anti-Aging. Molecules.

[43] Gozzi-Silva S.C., Teixeira F.M.E., Duarte A.J.D.S., Sato M.N., Oliveira L.D.M. (2021). Immunomodulatory Role of Nutrients: How Can Pulmonary Dysfunctions Improve?. Front. Nutr..

[44] Chen M., Sun Y., Wu Y. (2020). Lower Circulating Zinc and Selenium Levels Are Associated with an Increased Risk of Asthma: Evidence from a Meta-Analysis. Public Health Nutr..

[45] Zhang P. (2023). The Role of Diet and Nutrition in Allergic Diseases. Nutrients.

[46] Mazziotta C., Tognon M., Martini F., Torreggiani E., Rotondo J.C. (2023). Probiotics Mechanism of Action on Immune Cells and Beneficial Effects on Human Health. Cells.

[47] Winkler P., Ghadimi D., Schrezenmeir J., Kraehenbuhl J.-P. (2007). Molecular and Cellular Basis of Microflora-Host Interactions. J. Nutr..

[48] Michail S. (2009). The Role of Probiotics in Allergic Diseases. Allergy Asthma Clin. Immunol..

[49] Eslami M., Bahar A., Keikha M., Karbalaei M., Kobyliak N.M., Yousefi B. (2020). Probiotics Function and Modulation of the Immune System in Allergic Diseases. Allergol. Immunopathol..

[50] Niers L.E.M., Hoekstra M.O., Timmerman H.M., Van Uden N.O., De Graaf P.M.A., Smits H.H., Kimpen J.L.L., Rijkers G.T. (2007). Selection of Probiotic Bacteria for Prevention of Allergic Diseases: Immunomodulation of Neonatal Dendritic Cells. Clin. Exp. Immunol..

[51] Farhat Z., Scheving T., Aga D.S., Hershberger P.A., Freudenheim J.L., Hageman Blair R., Mammen M.J., Mu L. (2023). Antioxidant and Antiproliferative Activities of Several Garlic Forms. Nutrients.

[52] Zare A., Farzaneh P., Pourpak Z., Zahedi F., Moin M., Shahabi S., Hassan Z.M. (2008). Purified Aged Garlic Extract Modulates Allergic Airway Inflammation in BALB/c Mice. Iran. J. Allergy Asthma Immunol..

[53] Akhilender Naidu K., Thippeswamy N.B. (2002). Inhibition of Human Low Density Lipoprotein Oxidation by Active Principles from Spices. Mol. Cell. Biochem..

[54] Kempaiah R.K., Manjunatha H., Srinivasan K. (2005). Protective Effect of Dietary Capsaicin on Induced Oxidation of Low-Density Lipoprotein in Rats. Mol. Cell. Biochem..

[55] Zhang W., Zhang Y., Fan J., Feng Z., Song X. (2024). Pharmacological Activity of Capsaicin: Mechanisms and Controversies (Review). Mol. Med. Rep..

[56] Chen K.-S., Chen P.-N., Hsieh Y.-S., Lin C.-Y., Lee Y.-H., Chu S.-C. (2015). Capsaicin Protects Endothelial Cells and Macrophage against Oxidized Low-Density Lipoprotein-Induced Injury by Direct Antioxidant Action. Chem.-Biol. Interact..

[57] Dehzad M.J., Ghalandari H., Nouri M., Askarpour M. (2023). Antioxidant and Anti-Inflammatory Effects of Curcumin/Turmeric Supplementation in Adults: A GRADE-Assessed Systematic Review and Dose–Response Meta-Analysis of Randomized Controlled Trials. Cytokine.

[58] Mousa A.M., Alhumaydhi F.A., Abdellatif A.A.H., Abdulmonem W.A., AlKhowailed M.S., Alsagaby S.A., Al Rugaie O., Alnuqaydan A.M., Aljohani A.S.M., Aljasir M. (2022). Curcumin and Ustekinumab Cotherapy Alleviates Induced Psoriasis in Rats through Their Antioxidant, Anti-Inflammatory, and Antiproliferative Effects. Cutan. Ocul. Toxicol..

[59] Kaag S., Lorentz A. (2023). Effects of Dietary Components on Mast Cells: Possible Use as Nutraceuticals for Allergies?. Cells.

[60] Choi Y.-H., Yan G.-H., Chai O.H., Song C.H. (2010). Inhibitory Effects of Curcumin on Passive Cutaneous Anaphylactoid Response and Compound 48/80-Induced Mast Cell Activation. Anat. Cell Biol..

[61] Kong Z.-L., Sudirman S., Lin H.-J., Chen W.-N. (2020). In Vitro Anti-Inflammatory Effects of Curcumin on Mast Cell-Mediated Allergic Responses via Inhibiting FcεRI Protein Expression and Protein Kinase C Delta Translocation. Cytotechnology.

[62] Zeng J., Hao J., Yang Z., Ma C., Gao L., Chen Y., Li G., Li J. (2023). Anti-Allergic Effect of Dietary Polyphenols Curcumin and Epigallocatechin Gallate via Anti-Degranulation in IgE/Antigen-Stimulated Mast Cell Model: A Lipidomics Perspective. Metabolites.

[63] Haftcheshmeh S.M., Mirhafez S.R., Abedi M., Heydarlou H., Shakeri A., Mohammadi A., Sahebkar A. (2022). Therapeutic Potency of Curcumin for Allergic Diseases: A Focus on Immunomodulatory Actions. Biomed. Pharmacother..

[64] Mayo J.C., Sainz R.M. (2020). Melatonin from an Antioxidant to a Classic Hormone or a Tissue Factor: Experimental and Clinical Aspects 2019. Int. J. Mol. Sci..

[65] Rodriguez C., Mayo J.C., Sainz R.M., Antolín I., Herrera F., Martín V., Reiter R.J. (2004). Regulation of Antioxidant Enzymes: A Significant Role for Melatonin. J. Pineal Res..

[66] Maarsingh H., Zaagsma J., Meurs H. (2008). Arginine Homeostasis in Allergic Asthma. Eur. J. Pharmacol..

[67] Morris C.R., Makrides M., Ochoa J.B., Szajewska H. (2013). Arginine and Asthma. Nestlé Nutrition Institute Workshop Series.

[68] Wu G., Meininger C.J., McNeal C.J., Bazer F.W., Rhoads J.M., Wu G. (2021). Role of L-Arginine in Nitric Oxide Synthesis and Health in Humans. Amino Acids in Nutrition and Health.

[69] Sartorio M.U.A., Pendezza E., Coppola S., Paparo L., D’Auria E., Zuccotti G.V., Berni Canani R. (2021). Potential Role of Omega-3 Polyunsaturated Fatty Acids in Pediatric Food Allergy. Nutrients.

[70] Zeyda M., Säemann M.D., Stuhlmeier K.M., Mascher D.G., Nowotny P.N., Zlabinger G.J., Waldhäusl W., Stulnig T.M. (2005). Polyunsaturated Fatty Acids Block Dendritic Cell Activation and Function Independently of NF-κB Activation. J. Biol. Chem..

[71] Kong W., Yen J.-H., Vassiliou E., Adhikary S., Toscano M.G., Ganea D. (2010). Docosahexaenoic Acid Prevents Dendritic Cell Maturation and in Vitro and in Vivo Expression of the IL-12 Cytokine Family. Lipids Health Dis..

[72] Michaeloudes C., Abubakar-Waziri H., Lakhdar R., Raby K., Dixey P., Adcock I.M., Mumby S., Bhavsar P.K., Chung K.F. (2022). Molecular Mechanisms of Oxidative Stress in Asthma. Mol. Asp. Med..

[73] Paudel K.R., Dharwal V., Patel V.K., Galvao I., Wadhwa R., Malyla V., Shen S.S., Budden K.F., Hansbro N.G., Vaughan A. (2020). Role of Lung Microbiome in Innate Immune Response Associated with Chronic Lung Diseases. Front. Med..

[74] De Simoni E., Candelora M., Belleggia S., Rizzetto G., Molinelli E., Capodaglio I., Ferretti G., Bacchetti T., Offidani A., Simonetti O. (2024). Role of Antioxidants Supplementation in the Treatment of Atopic Dermatitis: A Critical Narrative Review. Front. Nutr..

[75] Anania C., Brindisi G., Martinelli I., Bonucci E., D’Orsi M., Ialongo S., Nyffenegger A., Raso T., Spatuzzo M., De Castro G. (2022). Probiotics Function in Preventing Atopic Dermatitis in Children. Int. J. Mol. Sci..

[76] Gardner K.G., Gebretsadik T., Hartman T.J., Rosa M.J., Tylavsky F.A., Adgent M.A., Moore P.E., Kocak M., Bush N.R., Davis R.L. (2020). Prenatal Omega-3 and Omega-6 Polyunsaturated Fatty Acids and Childhood Atopic Dermatitis. J. Allergy Clin. Immunol. Pract..

[77] Bousquet J., Anto J.M., Bachert C., Baiardini I., Bosnic-Anticevich S., Walter Canonica G., Melén E., Palomares O., Scadding G.K., Togias A. (2020). Allergic Rhinitis. Nat. Rev. Dis. Primers.

[78] Siddiqui Z., Walker A., Pirwani M., Tahiri M., Syed I. (2022). Allergic Rhinitis: Diagnosis and Management. Br. J. Hosp. Med..

[79] Ben-Shoshan M., Kanani A., Kalicinsky C., Watson W. (2024). Urticaria. Allergy Asthma Clin. Immunol..

[80] Saini S., Shams M., Bernstein J.A., Maurer M. (2020). Urticaria and Angioedema Across the Ages. J. Allergy Clin. Immunol. Pract..

[81] Galiniak S., Mołoń M., Biesiadecki M., Bożek A., Rachel M. (2022). The Role of Oxidative Stress in Atopic Dermatitis and Chronic Urticaria. Antioxidants.

[82] Jiang S., Hou Y., Meng L., Pu X., Zhu X., Tuo Y., Qian F., Mu G. (2021). Effect of *Lactiplantibacillus Plantarum* HM-22 on Immunoregulation and Intestinal Microbiota in α-Lactalbumin-Induced Allergic Mice. Food Funct..

[83] Di Costanzo M., Vella A., Infantino C., Morini R., Bruni S., Esposito S., Biasucci G. (2024). Probiotics in Infancy and Childhood for Food Allergy Prevention and Treatment. Nutrients.

[84] Fu L., Peng J., Zhao S., Zhang Y., Su X., Wang Y. (2017). Lactic Acid Bacteria-Specific Induction of CD^4+^Foxp^3+^T Cells Ameliorates Shrimp Tropomyosin-Induced Allergic Response in Mice via Suppression of mTOR Signaling. Sci. Rep..

[85] Mohseni A.H., Casolaro V., Bermúdez-Humarán L.G., Keyvani H., Taghinezhad-S S. (2021). Modulation of the PI3K/Akt/mTOR Signaling Pathway by Probiotics as a Fruitful Target for Orchestrating the Immune Response. Gut Microbes.

[86] Goldin J., Hashmi M.F., Cataletto M.E. (2025). Asthma. StatPearls.

[87] Allam V.S.R.R., Paudel K.R., Gupta G., Singh S.K., Vishwas S., Gulati M., Gupta S., Chaitanya M.V.N.L., Jha N.K., Gupta P.K. (2022). Nutraceuticals and Mitochondrial Oxidative Stress: Bridging the Gap in the Management of Bronchial Asthma. Environ. Sci. Pollut. Res..

[88] Xie Q., Yuan J., Wang Y. (2023). Treating Asthma Patients with Probiotics: A Systematic Review and Meta-Analysis. Nutr. Hosp..

[89] Harada T., Yamasaki A., Chikumi H., Hashimoto K., Okazaki R., Takata M., Fukushima T., Watanabe M., Kurai J., Halayko A.J. (2015). γ-Tocotrienol Reduces Human Airway Smooth Muscle Cell Proliferation and Migration. Pulm. Pharmacol. Ther..

[90] Adam-Bonci T.-I., Bonci E.-A., Pârvu A.-E., Herdean A.-I., Moț A., Taulescu M., Ungur A., Pop R.-M., Bocșan C., Irimie A. (2021). Vitamin D Supplementation: Oxidative Stress Modulation in a Mouse Model of Ovalbumin-Induced Acute Asthmatic Airway Inflammation. Int. J. Mol. Sci..

[91] Watkins S., Harrison T., Mushtaq S. (2024). A 12-Week Double-Blind Randomised Controlled Trial Investigating the Effect of Dietary Supplementation with 125 μg/d Vitamin D in Adults with Asthma. Br. J. Nutr..

[92] Jiang H., Yang G., Chen J., Yuan S., Wu J., Zhang J., Zhang L., Yuan J., Lin J., Chen J. (2024). The Correlation between Selenium Intake and Lung Function in Asthmatic People: A Cross-Sectional Study. Front. Nutr..

[93] Girdhar N., Kansal H., Garg K., Sharma S., Prabhu K.S., Chopra V., Tinkov A.A., Skalny A.V., Prakash N.T. (2022). Correlation of Serum Selenium in Asthma Patients with Severity of the Disorder. Biol. Trace Elem. Res..

[94] Abdelaziz I., Kotb M., Yassin N., Rabie W., Alsayed A., Hamed D. (2022). Zinc Supplementation Improves Nocturnal Asthma Symptoms. Pediatr. Sci. J..

[95] Sroka-Tomaszewska J., Trzeciak M. (2021). Molecular Mechanisms of Atopic Dermatitis Pathogenesis. Int. J. Mol. Sci..

[96] Frazier W., Bhardwaj N. (2020). Atopic Dermatitis: Diagnosis and Treatment. Am. Fam. Physician.

[97] Barta K., Fonacier L.S., Hart M., Lio P., Tullos K., Sheary B., Winders T.A. (2023). Corticosteroid Exposure and Cumulative Effects in Patients with Eczema. Ann. Allergy Asthma Immunol..

[98] Amestejani M., Salehi B.S., Vasigh M., Sobhkhiz A., Karami M., Alinia H., Kamrava S.K., Shamspour N., Ghalehbaghi B., Behzadi A.H. (2012). Vitamin D Supplementation in the Treatment of Atopic Dermatitis: A Clinical Trial Study. J. Drugs Dermatol..

[99] Lara-Corrales I., Huang C.M., Parkin P.C., Rubio-Gomez G.A., Posso-De Los Rios C.J., Maguire J., Pope E. (2019). Vitamin D Level and Supplementation in Pediatric Atopic Dermatitis: A Randomized Controlled Trial. J. Cutan. Med. Surg..

[100] Chang Y.-S., Lin M.-H., Lee J.-H., Lee P.-L., Dai Y.-S., Chu K.-H., Sun C., Lin Y.-T., Wang L.-C., Yu H.-H. (2016). Melatonin Supplementation for Children with Atopic Dermatitis and Sleep Disturbance: A Randomized Clinical Trial. JAMA Pediatr..

[101] Rakha A., Umar N., Rabail R., Butt M.S., Kieliszek M., Hassoun A., Aadil R.M. (2022). Anti-Inflammatory and Anti-Allergic Potential of Dietary Flavonoids: A Review. Biomed. Pharmacother..

[102] Alverina A.C., Susanto M. (2024). The Role of Polyphenols in Atopic Dermatitis: A Literature Review. World Nutr. J..

[103] Tian C., Feng Y., Chen T., Zhang Z., He X., Jiang L., Liu M. (2023). EGCG Restores Keratinocyte Autophagy to Promote Diabetic Wound Healing through the AMPK/ULK1 Pathway. Front. Biosci. (Landmark Ed.).

[104] Sun M., Luo J., Liu H., Xi Y., Lin Q. (2021). Can Mixed Strains of Lactobacillus and Bifidobacterium Reduce Eczema in Infants under Three Years of Age? A Meta-Analysis. Nutrients.

[105] Leermakers E.T.M., Sonnenschein-van Der Voort A.M.M., Heppe D.H.M., De Jongste J.C., Moll H.A., Franco O.H., Hofman A., Jaddoe V.W.V., Duijts L. (2013). Maternal Fish Consumption during Pregnancy and Risks of Wheezing and Eczema in Childhood: The Generation R Study. Eur. J. Clin. Nutr..

[106] Luo C., Peng S., Li M., Ao X., Liu Z. (2022). The Efficacy and Safety of Probiotics for Allergic Rhinitis: A Systematic Review and Meta-Analysis. Front. Immunol..

[107] Zajac A.E., Adams A.S., Turner J.H. (2015). A Systematic Review and Meta-analysis of Probiotics for the Treatment of Allergic Rhinitis. Int. Forum Allergy Rhinol..

[108] Peng Y., Li A., Yu L., Qin G. (2015). The Role of Probiotics in Prevention and Treatment for Patients with Allergic Rhinitis: A Systematic Review. Am. J. Rhinol. Allergy.

[109] Liu P., Hu T., Kang C., Liu J., Zhang J., Ran H., Zeng X., Qiu S. (2022). Research Advances in the Treatment of Allergic Rhinitis by Probiotics. J. Asthma Allergy.

[110] Iftikhar H., Awan M.O., Awan M.S., Mustafa K., Das J.K., Ahmed S.K. (2022). Role of Probiotics in Patients with Allergic Rhinitis: A Systematic Review of Systematic Reviews. Int. Arch. Otorhinolaryngol..

[111] Li Y., Cao Z., Guo J., Li Q., Su J. (2021). Effects of Serum Vitamin D Levels and Vitamin D Supplementation on Urticaria: A Systematic Review and Meta-Analysis. Int. J. Environ. Res. Public Health.

[112] Piotin A., Oulehri W., Charles A.-L., Tacquard C., Collange O., Mertes P.-M., Geny B. (2024). Oxidative Stress and Mitochondria Are Involved in Anaphylaxis and Mast Cell Degranulation: A Systematic Review. Antioxidants.

[113] Wang J., Zhang Y., Hu S., Ge S., Jia M., Wang N. (2021). Resveratrol Inhibits MRGPRX2-Mediated Mast Cell Activation via Nrf2 Pathway. Int. Immunopharmacol..

[114] Civelek M., Bilotta S., Lorentz A. (2022). Resveratrol Attenuates Mast Cell Mediated Allergic Reactions: Potential for Use as a Nutraceutical in Allergic Diseases?. Mol. Nutr. Food Res..

[115] Shaik Y., Caraffa A., Ronconi G., Lessiani G., Conti P. (2018). Impact of Polyphenols on Mast Cells with Special Emphasis on the Effect of Quercetin and Luteolin. Central Eur. J. Immunol..

[116] Ding Y., Che D., Li C., Cao J., Wang J., Ma P., Zhao T., An H., Zhang T. (2019). Quercetin Inhibits Mrgprx2-Induced Pseudo-Allergic Reaction via PLCγ-IP3R Related Ca^2+^ Fluctuations. Int. Immunopharmacol..

[117] Dabbaghzadeh A., Ghaffari J., Moradi S., Sayadian separghan D. (2023). Probiotics on Chronic Urticaria: A Randomized Clinical Trial Study. Casp. J. Intern. Med..

[118] Villegas B.V., Benitez-del-Castillo J.M. (2021). Current Knowledge in Allergic Conjunctivitis. Turk. J. Ophthalmol..

[119] Portnoy J.M., Dinakar C. (2004). Review of Cetirizine Hydrochloride for the Treatment of Allergic Disorders. Expert Opin. Pharmacother..

[120] Ciprandi G., Ricca V., Paola F., Alessandra B., Riccio A.M., Milanese M., Frati F., Tosca M.A. (2004). Duration of Antiinflammatory and Symptomatic Effects after Suspension of Intranasal Corticosteroid in Persistent Allergic Rhinitis. Eur. Ann. Allergy Clin. Immunol..

[121] Marseglia G., Licari A., Ciprandi G. (2020). Complementary Treatment of Allergic Rhinoconjunctivitis: The Role of the Nutraceutical Lertal(R). Acta Biomed. Atenei Parm..

[122] Yu H., Qiu J.-F., Ma L.-J., Hu Y.-J., Li P., Wan J.-B. (2017). Phytochemical and Phytopharmacological Review of *Perilla frutescens* L. (Labiatae), a Traditional Edible-Medicinal Herb in China. Food Chem. Toxicol..

[123] Ariano R. (2015). Efficacy of a Novel Food Supplement in the Relief of the Signs and Symptoms of Seasonal Allergic Rhinitis and in the Reduction of the Consumption of Anti-Allergic Drugs. Acta Biomed..

[124] Cosme-Blanco W., Arroyo-Flores E., Ale H. (2020). Food Allergies. Pediatr. Rev..

[125] Antunes M.M., Coelho B.S.L., Vichi T.M., Santos E.A.D., Gondim F.K.B., Diniz A.B., Aguilar E.C., Cara D.C., Porto L.C.J., Castro I.C.D. (2019). Oral Supplementation with Capsaicin Reduces Oxidative Stress and IL-33 on a Food Allergy Murine Model. World Allergy Organ. J..

[126] Di Salvo E., Gangemi S., Genovese C., Cicero N., Casciaro M. (2023). Polyphenols from Mediterranean Plants: Biological Activities for Skin Photoprotection in Atopic Dermatitis, Psoriasis, and Chronic Urticaria. Plants.

